# Comparative Analysis of Heart Regeneration: Searching for the Key to Heal the Heart—Part I: Experimental Injury Models to Study Cardiac Regeneration

**DOI:** 10.3390/jcdd10080325

**Published:** 2023-07-31

**Authors:** Juan Manuel Castillo-Casas, Sheila Caño-Carrillo, Cristina Sánchez-Fernández, Diego Franco, Estefanía Lozano-Velasco

**Affiliations:** 1 Cardiovascular Development Group, Department of Experimental Biology, University of Jaén, 23071 Jaén, Spain; jmcasas@ujaen.es (J.M.C.-C.); scano@ujaen.es (S.C.-C.); csfernan@ujaen.es (C.S.-F.); dfranco@ujaen.es (D.F.); 2 Medina Foundation, 18007 Granada, Spain

**Keywords:** cardiac disease, myocardial infarction, molecular pathways, hypoxia, metabolism, inflammation, cell cycle, fibrosis, heart regeneration

## Abstract

Cardiovascular diseases are the leading cause of death worldwide, among which, ischemic heart disease is the most prevalent. Myocardial infarction results from occlusion of a coronary artery, which leads to an insufficient blood supply to the myocardium. As is well known, the massive loss of cardiomyocytes cannot be solved due the limited regenerative ability of the adult mammalian heart. In contrast, some lower vertebrate species can regenerate the heart after injury; their study has disclosed some of the involved cell types, molecular mechanisms and signaling pathways during the regenerative process. In this two-part review, we discuss the current state of the principal response in heart regeneration, where several involved processes are essential for full cardiac function in recovery.

## 1. Introduction

Cardiovascular diseases are the leading cause of death worldwide, and, among all of them, ischemic heart disease affects around 126 million individuals [1,2]. Myocardial Infarction (MI) is driven by death and loss of cardiomyocytes (CMs) at the site of ischemic injury [3]. The lack of oxygen supply leads to adverse remodeling in the affected myocardium leading to cell death. Moreover, the restoration of the oxygenated blood flow during the reperfusion process can paradoxically accelerate an additional myocardial injury due to a high production of reactive oxygen species (ROS), promoting oxidative stress and thus an extra wave of CM cell deaths [4,5,6,7]. Necrotic CMs are replaced by myofibroblasts forming a fibrotic tissue scar that drastically diminishes CMs’ contractile potential affecting the functional rate of the heart and eventually leading to heart failure (HF) [8,9,10,11]. Mammalian adult hearts do not have the ability to survive a substantial loss of CMs, making cardiac regeneration one of the major avenues in human cardiovascular research [12,13]. This review has as its main objective providing an in-depth analysis of the different animal models used for the study of cardiac regeneration, summarizing the main molecular targets and related signaling pathways involved in MI and the regeneration process.

## 2. Experimental Models of Cardiac Regeneration

Cardiac regeneration is an ancestral trait in vertebrates, a general capacity that seems to be inversely correlated with evolutionary complexity across the animal kingdom [14,15]. For example, the human heart has a very limited capacity for cardiac regeneration in contrast to fish and amphibian organisms [16,17]. Some years ago, Field’s lab evidenced that a low percentage of mouse ventricular CMs have proliferative capacity in normal conditions in mice, becoming higher after injury [18]. In the same line, it has been demonstrated that 1% of human CMs are renewed each year [12]. In a cardiac injured scenario, this low percentage of CM renewal is not enough to repair the damaged myocardium. Some laboratories, within the cardiovascular field, are focusing their efforts on increasing the proliferative ability of CMs in order to enhance the regenerative capacity of mammalian hearts. To better understand the biology of MI, as well as to develop different therapeutic strategies, in vitro, ex vivo and in vivo models have been developed. 

The first approach is the in vitro cardiac model which implies primary cultures and/or cell lines. Concretely, CMs can be obtained from animal or human hearts or by the differentiation process from stem cells. In general terms, CMs from primary culture have some limitations for the study of cardiac physiology, such as low proliferative capacity and the absence of spontaneous beat, among others [19,20,21,22,23]. As an alternative, differentiated CMs derived from pluripotent cells, mesenchymal stem cells (MSCs), embryonic stem cells (ESCs) or induced pluripotent stem cells (iPSCs), are essential for the development of MI models [24,25,26,27,28]. Considering in vitro experiments, two-dimensional (2D) models have some limitations; concretely, they do not take into consideration the relationship between CMs and other tissues or, even more importantly, the immune and/or paracrine systems, and are far away from reproducing in vivo conditions [29,30,31]. In contrast, three-dimensional (3D) models have the ability to create conditions more similar to in vivo situations, i.e., CMs interaction with immune, endothelial and stromal cells, as well as with the extracellular matrix (ECM) [26,27,28]. The main limitation of 3D models is the absence of blood irrigation to assess an adequate supply of nutrients and oxygen [32,33]. However, in general terms, in vitro models enable the possibility of bringing cells under different controlled conditions, alone or combined, which lead to single-cell analysis or to the study of cell–cell interactions by microscopy and other protein techniques which are restricted to in vitro cultures [34,35]. 

On the other hand, ex vivo models involve keeping the heart outside of the body in a normal or retrograde perfusion system. This kind of model offers the opportunity to generate ischemic and reperfusion situations to analyze the effects of MI [36,37,38]. The main advantage of this model is that the infarct size can be measured and left ventricular function can be easily assessed [35,39,40]. Moreover, this model can be ideal for drug screening and to study interventions for protective properties [35]. Nevertheless, similarly to the in vitro models, there is no relation with other tissues and/or systems, and, moreover, one must consider that there are other associated variables that can interfere with observations: for example, tissue stability, source of energy and edema risk [35]. 

Last but not least are the in vivo models: they represent the most used model to test MI effects as well as for drug and safety studies. This model offers the opportunity of analyzing the vast majority of physiological changes generated in response to MI, i.e., inflammatory processes and scar formation as well as the possibility of identifying blood biomarkers [35]. In the in vivo scenario, researchers have the possibility of employing different strategies that enable the study of different molecular mechanisms, but need to take into consideration the variability associated to injury degree (mild, moderate and severe), as well as surgical and postoperative mortality [35]. As it is well known some organisms are able to regenerate cardiac damage achieving full anatomical and functional recovery, for example amphibians, newts, axolotls and zebrafish [41,42,43,44]. However, mammalian hearts have only a tiny window of regenerative capacity after birth [45,46,47], therefore, as can be expected, there are some differences which are responsible for the achievement, or not, of adequate regeneration. These aspects will be analyzed in the forthcoming paragraphs.

## 3. Injury Models to Study Cardiac Healing

Nowadays, the most important goal of cardiac researchers is to decipher the mechanisms that control heart regeneration in distinct animal models with the intention of stimulating cardiac repair in adult mammals. To perform this challenging goal, several different methods are applied to simulate a cardiac injury that is similar to the damage generated by MI. 

**Figure 1:**
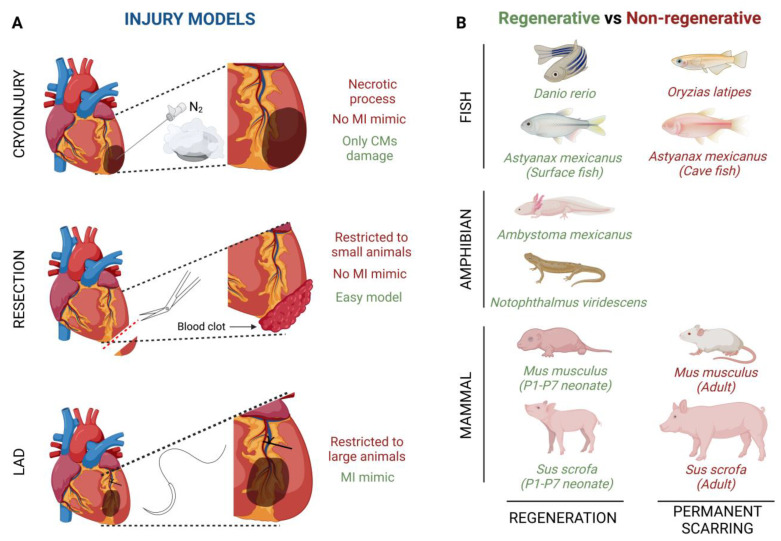
Graphical summary of (**A**) injury models of myocardial infarction and (**B**) interspecies comparisons of heart regenerative and non-regenerative capacity.

The cryoinjury technique is based on the exposition of the cardiac apical left ventricle area to a liquid nitrogen cooled cryoprobe [48] (Figure 1A). By using this technique, it is noteworthy that, depending on the damage severity, the cardiac regenerative response can be different. Transmural injury is the highest degree of damage with this technique, where the full wall diameter of the ventricle is affected, whereas non-transmural injury, where the cryoprobe does not penetrate the wall ventricle, inflicts mild damage [49]. This cryoinjury method implies a necrotic process whose side effects do not fully mimic the ischemic characteristics developed in a human heart. The main advantage of this method is that cryothermia is capable of inflicting damage only to CMs, preserving tissue collagen, since both cardiac fibroblasts (CFs) and collagen are resistant to cryoinjury [50].

Another injury model is apical resection; it consists of the removal of a small piece of tissue, no more than 15%, from the apical left ventricle wall after heart exposure [51,52] (Figure 1A). Immediately after apical resection there is an inflammatory process which ends with the generation of a blood clot that seals the resected area, thus starting the regenerative process [46]. Although apical resection provides an easy model of CM loss, the main limitation of this procedure is restricted to P1-P7 mice because in older animals the mortality rate is much more elevated [51]. Like cryoinjury, apical resection does not fully capture the ischemic damage of a MI.

Finally, left anterior descending coronary artery (LAD) ligation is considered the best technique to mimic an MI ischemic injury (Figure 1A). After heart exposure, the LAD is ligated with one single stitch and no blood flow is present in the area, while the ventricular myocardium is not affected [53]. Although this technique completely captures the pathobiological and pathophysiological characteristics of cardiac infarction, it has usually been used in adult mice or larger animals, since its feasibility in zebrafish or neonatal mice is limited by small size or a less obvious coronary vasculature [54,55]. 

## 4. Heart Regeneration after Injury in Different Animal Models

One of the major challenges for cardiac researchers and clinicians is the identification of the combinatorial response exerted by CFs, endothelial cells (ECs) and CMs, among other cell types, to mitigate the cardiac damage after MI. In order to be able to measure morphological, functional and molecular parameters during the MI and cardiac regeneration trajectory, in vivo animal models are needed to run through the pathological effects that MI exerts over the cardiac muscle. 

### 4.1. Invertebrate Models

The ability to replace lost body parts or tissues is a phenomenon that is peculiar to a few organisms from different clades in the animal kingdom. Within the invertebrate group, hydras and planarians stand out, due to their ability to completely regenerate their bodies after an amputation [56]. These models allow us a first approach to the morphological and cellular dynamic changes involved in regeneration [57]. Hydras and planarians are two in vivo models with simple cellular organization that make them useful model systems to better study and understand stem cell behavior and regenerative processes in higher evolutionary models.

### 4.2. Vertebrate Models 

Heart regeneration is observed in many fishes and amphibians as well as in fetal and early neonatal mammals [15], as described in the following paragraphs.

- Fish models

Several fish species have shown the ability to repair an injured heart through the induction of CM proliferation (Figure 1B). For example, zebrafish (*Danio rerio*) have the ability of total heart regeneration 60 days after ~20% apical resection of the ventricle, by activating the proliferation rate of the CMs present in the surrounding injured area [16,58]. Something similar happens when ~25% of the zebrafish’s ventricle is damaged by using a cryoprobe. In this type of heart injury model, although the regeneration process is much slower, the heart achieves restoration in approximately ~180 days [59,60,61]. Finally, Poss’ lab developed a tamoxifen-inducible genetic ablation model that allowed the promotion of 60% of CM cell death. In this case, 30-day post tamoxifen induction they observed a complete re-muscularized ventricle [62]. Moreover, a few years ago, Gonzalez-Rosas et al. (2018) [63] reported that most of the zebrafish’s CMs contain only two sets of homologous chromosomes. These diploid CMs have the ability to proliferate, inducing heart regeneration [64]. Finally, considering that zebrafish resides in a hypoxic environment, this restrictive O_2_ condition enables CM dedifferentiation and proliferation leading to heart regeneration [58]. Another fish species with regenerative capacity is the *Astyanax mexicanus*, which, after a spring flooding millions of years ago, diverged into cave-dwelling and surface populations [65,66,67] (Figure 1B). Studies carried out in these species by Stockdale et al. (2018) reported that surface fishes are able to achieve full heart recovery after ventricular apical resection, whereas cave-dwelling fishes, from Pachón Cave, develop a permanent fibrotic scar; they, thus, lost their ability to regenerate the heart [68]. Of significance, both *Astyanax mexicanus* surface and cave-dwelling populations promote CM proliferation in the surrounding injured area, similar to zebrafish. However, Pachón Cave fishes have lower expression of a leucine-rich repeat containing 10 (lrrc10) than surface fishes after injury, indicating the needed for upregulation of lrrc10 in complete heart regeneration [68]. Finally, the medaka fish (*Oryzias latipes*) is not able to achieve heart regeneration (Figure 1B). In contrast to Pachón Cave fishes, medaka CMs do not proliferate after heart injury and moreover they lack expression of endocardial retinoic acid (RA)-synthesizing enzyme (Raldh2), which is essential for the stimulation of CM proliferation in zebrafish [69,70].

- Amphibian models

Similar to fishes, some urodele amphibians have the ability to regenerate several tissues and organs [71,72] (Figure 1B). For example, axolotls (*Ambystoma mexicanus*), which are neotenic aquatic urodele amphibians, are capable of regenerating the heart after injury, resection and cryo-injury, but only in the larval phase [71,72,73]. However, when axolotls undergo metamorphosis and get into the adulthood stage, regeneration, in general terms, is reduced and some morphological defects are still observed [74]. This animal group loses its overall regenerative capacity as it undergoes metamorphosis, concomitantly with maturation of the immune system, which is comparable to that of mammals [75,76]. There are no studies about heart regeneration in adult axolotls, however it is important to highlight that thyroid hormone, which is necessary in this species to undergo metamorphosis into a land-dwelling adult, impairs heart regeneration in zebrafish [73,77]. In these terms, newts as *Notophthalmus viridescens*, bear heart regeneration capacity dependent on the type of injury [17,42,78,79,80] (Figure 1B). It has been demonstrated that there is a limited CM dedifferentiation and proliferation in the CMs present in the wounded area, and, moreover, ECM component deposition, such as collagen III, is observed just before reconstitution of the myocardium [42,80,81]. Finally, although salamanders are capable of regenerating their limbs, no evidence has been shown in terms of cardiac regeneration [82].

- Chicken models

Avian cardiac regenerative capacity has not been thoroughly investigated. Burn lesions in chicken (*Gallus gallus*) myocardium resolve as regeneration in 3- and 5-day old chick embryos [83,84]. Furthermore, a study carried out by Novikov and Khloponin in 1984 demonstrated that chickens have the ability to repair cardiac damage at early embryonic stages. This process takes about 7 to 10 days after injury with the intervention of all three layers of the embryonic heart, i.e., epicardium, endocardium and myocardium [84]. If the cardiac injury takes place in 18-day-old chick embryos or hatched chickens, cardiac regeneration is not achieved and scar tissue is formed [84].

- Mammal models

Lastly, it is widely known that the mammalian heart does not have the ability to regenerate after an injury process (Figure 1B). However, more than a decade ago, Porrello et al. (2011), revealed that heart regeneration was achieved in mice (*Mus musculus*) when apical resection was performed at postnatal day 1 (P1). This regenerative event lasted a period of 21 days and was carried out by proliferation of the existing CMs in the surrounding injured area [45,85]. Similarly, a full cardiac recovery is observed in mice after ligation of the left anterior descending (LAD) coronary artery in P1 [86]. On the other hand, the heart behavior after ventricle cryoinjury is different depending on severity; i.e., non-transmural cryoinjury in P1 mice undergoes healing, while P1 mice with transmural cryoinjury do not attain complete regeneration [49,87,88]. Something similar happens with ventricle apical resection, indicating that large injured areas restrict heart regeneration [89]. Several decades ago, it was evidenced that the mouse heart loses its regenerative potential after one week of life; however, this regenerative window is a bit controversial, as some authors indicate that cardiac regeneration decreases as soon as 48 h (P2) after a fibrotic response after injury can be observed [45,90]. It is noteworthy that significant changes occur in mice after birth, such as the transition from a hypoxic environment during embryonic development to an oxygenated state at P1, which leads to CM cell cycle arrest and thus promotes a non-regenerative state [91]. This time window coincides with the developmental polyploidization of the neonatal myocardium, which is proposed to be a major barrier to cardiac regeneration in mice [92,93]. During the regenerative response in mice, similarly to fishes and axolotls, the neonatal mouse injury response is initiated by rapid clotting, inflammatory cell infiltration into the injured area, epicardial activation and CM proliferation [86]. Similarly, the neonatal regenerative potential is present in large mammal such as the neonatal pig (*Sus Scrofa*) (Figure 1B). A few years ago, Ye et al. (2018) [46] observed a regenerated cardiac muscle and fully functional recovery after permanent LAD ligation in P2 pigs; however, the same injury in P14 pigs ended in a fibrotic scar, thin myocardium and a dysfunctional heart [46]. Finally, although there are limited studies about heart regeneration in neonatal humans (*Homo sapiens*), some cases of massive myocardial infarctions shortly after birth without long-term deficits have been documented. However, as in rodents, post-infarct CM proliferation induction in adult humans is limited and cardiac injury resolution consists of permanent fibrosis and loss of cardiac output [94,95,96]. Some labs have evidenced that newborn children have the ability to achieve complete myocardial function recovery after massive cardiogenic shock in MI, thrombolytic occlusion of the proximal LAD and some congenital heart diseases [97,98,99]. Similar to other species, young humans’ CM proliferation helps to improve cardiac regeneration [12,100].

## 5. Conclusions and Perspectives

Myocardial infarction is a major clinical burden worldwide and therefore biomedical strategies to heal the injured heart represent an unmet clinical priority [2,3]. Distinct experimental models of cardiac injury have been established, ranging from cell cultures to in vivo MI surgical procedures. Similarly, distinct models of cardiac injury have been implemented such as from ventricular resection, superficial vs transmural cryoinjury, transaortic constriction and left coronary artery occlusion. Each of these experimental and cardiac injury models can provide answers to specific questions, having, in all cases, advantages and disadvantages. 

Interestingly, innate cardiac regenerative potential varies along the evolutionary scale in vertebrates, providing additional biological working models to dissect the cellular and molecular bases of injury healing [10,11,12,13,14,15,16,17,18]. Over the last decade, a large body of efforts have been devoted to performing comparative analyses of cardiac injury and regeneration in those species with different regenerative capacities, unravelling the determinant role of the inflammatory response, extracellular remodeling and cardiomyocyte ploidy in the resolution of the injured heart. Such evidence demonstrated a complex temporal and cellular interplay between distinct cardiovascular cell types that critically compromise the final outcome [41,42,43,44,45,46,47,48,49]. While consistent differences in the span of the inflammatory response and subsequent scar resolution have been identified in different regenerative vs non-regenerative species, it remains to be fully established how universal are those findings in different vertebrate species. A more detailed analysis of the distinct molecular pathways involved in cardiac regeneration is provided in part II of this review.

## Data Availability

No new data generated.
